# Survey of Autonomous Vehicles’ Collision Avoidance Algorithms

**DOI:** 10.3390/s25020395

**Published:** 2025-01-10

**Authors:** Meryem Hamidaoui, Mohamed Zakariya Talhaoui, Mingchu Li, Mohamed Amine Midoun, Samia Haouassi, Djamel Eddine Mekkaoui, Abdelkarim Smaili, Amina Cherraf, Fatima Zahra Benyoub

**Affiliations:** 1 School of Software Technology, Dalian University of Technology, Dalian 116024, China; meryem@dlut.edu.cn (M.H.); mingchul@dlut.edu.cn (M.L.); smaili97@mail.dlut.edu.cn (A.S.); 2 School of Control Science and Engineering, Dalian University of Technology, Dalian 116024, China; talhaouizakariya@dlut.edu.cn; 3 School of Computer and Information Engineering, Jiangxi Normal University, Nanchang 330022, China; 4 School of Computer Science and Technology, Dalian University of Technology, Dalian 116024, China; souma@mail.dlut.edu.cn (S.H.); mekdjamel@mail.dlut.edu.cn (D.E.M.); 5 School of Mathematics, Abou-Bakr Belkaid University, Tlemcen 13000, Algeria; amina.cherraf@univ-tlemcen.dz; 6 School of Automation and Electrical Engineering, Beihang University, Beijing 100191, China; fatima.beny@buaa.edu.cn

**Keywords:** collision avoidance, autonomous vehicles, path planning, sensor-based approaches, decision-making, machine learning

## Abstract

Since the field of autonomous vehicles is developing quickly, it is becoming increasingly crucial for them to safely and effectively navigate their surroundings to avoid collisions. The primary collision avoidance algorithms currently employed by self-driving cars are examined in this thorough survey. It looks into several methods, such as sensor-based methods for precise obstacle identification, sophisticated path-planning algorithms that guarantee cars follow dependable and safe paths, and decision-making systems that allow for adaptable reactions to a range of driving situations. The survey also emphasizes how Machine Learning methods can improve the efficacy of obstacle avoidance. Combined, these techniques are necessary for enhancing the dependability and safety of autonomous driving systems, ultimately increasing public confidence in this game-changing technology.

## 1. Introduction

The concept of autonomous driving is gaining increasing interest and investment from scholars, industry experts, and policymakers [1,2]. This technology has the potential to transform transportation by enabling vehicles to navigate and operate without human intervention [3]. The rapid advancements in autonomous vehicle technologies highlight the pressing need for reliable collision avoidance systems to ensure safe and efficient navigation [4]. Developing robust collision avoidance algorithms is crucial for establishing secure and effective transportation systems.

Many researchers are dedicated to advancing highly effective collision avoidance methods for autonomous vehicles. Their commitment arises from a shared goal to enhance the safety and reliability of self-driving technologies, ultimately establishing autonomous vehicles as a widely trusted and adopted mode of transportation. Consequently, numerous valuable approaches have been proposed. Several comprehensive review papers have thoroughly evaluated and assessed various collision avoidance techniques.

Fu et al. [5] explore data-driven collision avoidance methods, focusing on sensing, vehicular communications, and AI-based algorithms to improve driving safety. In a separate study, Tijani [6] investigates obstacle detection and avoidance systems for autonomous vehicles, aiming to enhance security and efficiency in self-driving technology. Additionally, Dahl et al. [7] conduct a literature review and analysis of threat-assessment approaches used in intelligent vehicle systems for collision avoidance.

Other surveys focus on strategies for multiple-vehicle collision avoidance (MVCA) in automated and unmanned systems. Md Muzahid et al. [8] review MVCCA (multiple vehicle cooperation and collision avoidance) techniques and challenges, proposing an AI-enabled framework for automated vehicles. Liu and Bucknall [9] concentrate on cooperative motion planning and control for multi-vehicle systems across various domains, emphasizing adaptable formation strategies for collision avoidance.

Finally, in their study, the authors of [10,11,12,13] examined the complex nature of road traffic, which involves vehicles, roads, and various road users, such as pedestrians. They specifically investigated the potential of autonomous cars to avoid collisions with pedestrians.

While prior surveys provide valuable insights into specific aspects of collision avoidance, such as sensor types, trajectory planning, or decision-making techniques, this study offers a more comprehensive analysis. Unlike these works, we provide an integrated review of collision avoidance algorithms, categorizing them into sensor-based approaches, path planning, decision-making strategies, and Machine Learning techniques. This broader perspective enables readers to understand the interplay between these methodologies and their real-world applicability. In addition to expanding the scope of existing reviews, our work distinguishes itself by addressing real-world challenges faced by autonomous ground vehicles (AGVs) and highlighting the integration of cross-domain technologies, such as edge computing, V2X communication, and virtual reality. These technologies offer innovative solutions to current limitations and align with the unique demands of AGVs. Moreover, we synthesize data from multiple sources, reviewing the top literature from 2020 to 2024, making this study a practical and up-to-date resource for autonomous vehicle experimenters. The fundamental advantages of this manuscript are summarized as follows:

1. This paper comprehensively surveys various collision avoidance algorithms for autonomous vehicles (AVs), encompassing methodologies like sensor-based approaches, path planning, decision-making strategies, and machine-learning techniques.
2. A detailed comparative analysis is presented, highlighting each methodology’s strengths, limitations, and real-world applicability, enabling readers to grasp the advantages and disadvantages of different approaches quickly.
3. This survey identifies current projects and challenges while suggesting potential areas for future research to improve collision avoidance algorithms.
4. Unlike UAV-focused reviews, such as Lu et al. [14] and Huang et al. [15], this study targets AGVs, addressing ground-specific challenges like urban navigation, pedestrian unpredictability, and adverse weather conditions.

This survey is organized as follows: Section 2 discusses the sensor technology in collision avoidance. Section 3 provides an introduction to various technologies and techniques used for collision avoidance in autonomous vehicles. Section 4 introduces some real-life applications and challenges. Finally, Section 5 presents the future directions and conclusion.

**Figure 1:**
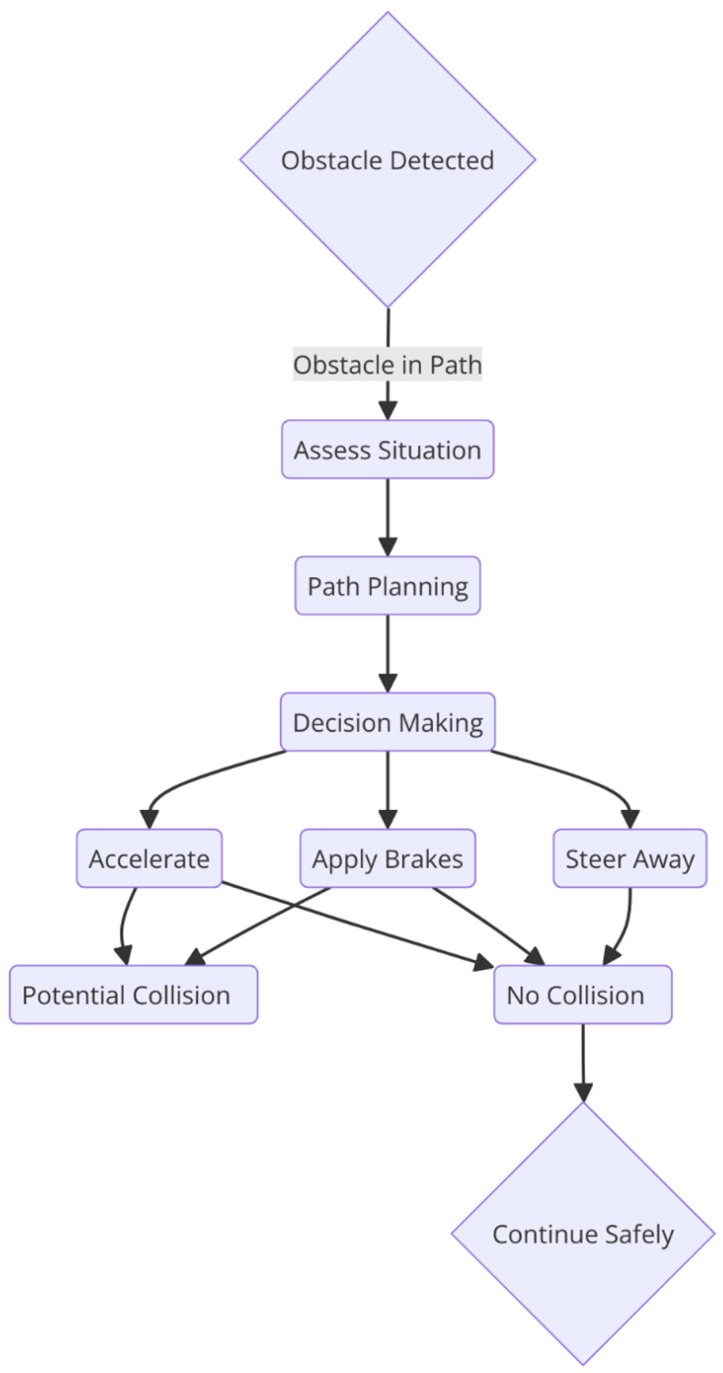
Framework of collision avoidance algorithm.

Autonomous vehicles must have collision avoidance systems to prevent accidents and ensure safety. These systems rely on sensors, algorithms, and communication technology to detect and respond to potential collision risks. By proactively identifying and evading obstacles, they help autonomous vehicles navigate safely, as shown in Figure 1. Implementing robust collision avoidance systems is crucial for gaining public trust, improving road safety, and enabling the widespread adoption of autonomous vehicles. There are various technologies and methods used for collision avoidance in autonomous vehicles.

## 2. Sensor Technologies in Collision Avoidance

**Figure 2:**
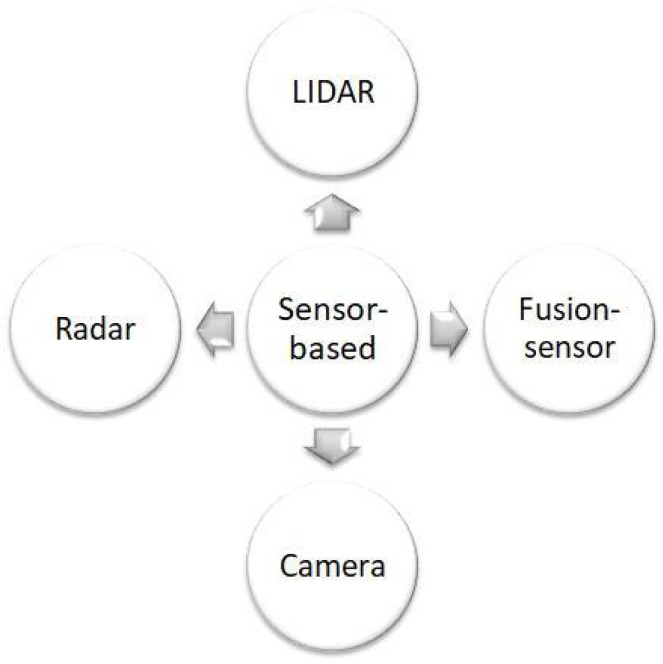
Framework sensor-based approaches.

The rapid advancement of autonomous vehicle technology underscores the importance of sensor-based approaches for reliable perception and decision-making Figure 2. Sensors such as cameras, radar, and LiDAR are indispensable for detecting obstacles, estimating distances, and providing environmental context. While each sensor type has unique strengths, they also face limitations in specific scenarios (e.g., adverse weather or dynamic environments). To address these challenges, sensor fusion has emerged as a powerful approach, combining the complementary strengths of different sensors to enhance accuracy, reliability, and robustness in real-time collision avoidance systems. In this section, we cite the papers that show the best performance in this field and give a detailed description in Table 1.

### 2.1. Camera

Cameras are essential for collision avoidance in autonomous vehicles, acting as line-of-sight (LOS) sensors that detect obstacles and other cars. They benefit from recognizing objects such as pedestrians, traffic signs, and lane markings. In [16], a monocular camera with visible light communication improves V2V (vehicle-to-vehicle) positioning accuracy using the taillight as a baseline and a Kalman Filter (KF) to reduce noise. The RGB camera used was the Sony IMX219, manufactured by Sony Corporation, Tokyo, Japan. The Kalman Filter (KF) algorithm was implemented using MATLAB R2022a (MathWorks, Natick, MA, USA). In contrast, Cabon et al. [17] apply a CNN (convolutional neural network) for position and motion estimation. However, it struggles to distinguish different traffic types. Data processing was performed using TensorFlow v2.11 (Google LLC, Mountain View, CA, USA).

Mallik et al. [18] combine a monocular RGB camera and RetinaNet for real-time object detection, achieving 24 FPS (frames per second) The cameras used were Sony IMX477 models, also from Sony Corporation, Tokyo, Japan. Rill and Farago predict Time-to-Collision (TTC) using monocular vision and YOLO-based (You Only Look Once) object detection [19]. The YOLO v5 [https://github.com/ultralytics/yolov5, , accessed on 7 January 2025] model was implemented for object detection and integrated with OpenCV v4.5, developed by Intel Corporation, Santa Clara, CA, USA. Additionally, Jao et al. [20] improve vehicle localization by matching real-time images to a pre-built database. The monocular RGB cameras used were Sony IMX219, manufactured by Sony Corporation, Tokyo, Japan, and the data were processed using OpenCV v4.5 (Intel, Santa Clara, CA, USA). Also, using 3D detection techniques, Zhe et al. [21] introduce a method for calculating inter-vehicle distance using a monocular camera system. These systems incorporated TensorFlow v2.11 (Google LLC, Mountain View, CA, USA) for machine learning-based enhancements. While this approach works well for detecting objects, it has difficulty distinguishing between different types of traffic, such as cars, trucks, or motorcycles, which can affect the detection accuracy.

### 2.2. Radar

Radar uses radio waves to detect objects by measuring how long it takes for the waves to bounce back. It works well in all weather conditions, including rain, snow, and fog, where cameras and LiDAR might have trouble. Muckenhuber et al. [22] presents a radar solution for car detection in ADAS (advanced driver assistance systems) systems. The ARS408-21 radar sensor used was manufactured by Continental AG, Hanover, Germany. Radar data analysis was performed using MATLAB 2022a (MathWorks, Natick, MA, USA).

Sohail et al. [23] leverage Machine Learning with radar data to enhance vehicle position estimation, outperforming the other sensor-based approaches, particularly in adverse weather conditions. Data processing was conducted using Python libraries, including Scikit-learn v1.3.0 and PyTorch v2.0.

Additionally, Choi et al. [24] propose a weighted interpolation model to improve radar accuracy for vehicle tracking, addressing uncertainty and latency issues, the implementation using Python v3.9 and NumPy v1.21. Srivastav and Mandal [25] highlight the potential of radar and deep learning to improve autonomous vehicle safety but note challenges in data resolution. Radar is commonly used for adaptive cruise control and collision warnings, helping to detect obstacles from a distance. Their research utilized ARS408-21 radar sensors from Continental AG, Hanover, Germany, with radar data processed in MATLAB 2022a (MathWorks, Natick, MA, USA).

### 2.3. LiDAR (Light Detection and Ranging)

This technology is widely used in the research community for accurate vehicle position estimation [26]. For instance, Dazlee et al. [27] examined a LiDAR-based approach for obstacle detection in VANETs, utilizing the Velodyne HDL-32E sensor, manufactured by Velodyne Lidar, Inc., San Jose, CA, USA, comparing two variants of YOLO, Complex YOLO and Tiny YOLO, which utilize LiDAR data. Complex YOLO exhibited significantly higher average precision, with room for further improvement through enhanced hardware.

Moreover, Saha et al. [28] present methods utilizing LiDAR-generated 3D point clouds for detecting and tracking obstacles, contributing to real-time navigation and dynamic collision prevention. Utilizing ROS Noetic Ninjemys (Open Robotics, Mountain View, CA, USA) for processing and integration.

The review paper [29] underscores LiDAR’s role in collision detection and avoidance for autonomous driving, examining recent advancements and challenges. LiDAR provides accurate data for obstacle detection and vehicle positioning, but it can be expensive and may have reduced performance in poor weather conditions.

### 2.4. Fusion Sensor

Sensor fusion is valuable for collision avoidance in autonomous vehicles. It combines sensors to enhance tracking accuracy and object detection for real-time decision-making. Guan et al. [30] propose a camera-and-LiDAR-based method that integrates 2D LiDAR data with YOLO detection. The LiDAR sensor was the Velodyne VLP-16, and the cameras were Sony IMX477 models, both integrated using NVIDIA DriveWorks SDK v3.5 (NVIDIA Corporation, Santa Clara, CA, USA). Kotur et al. [31] propose a method combining LiDAR and camera data for enhanced tracking and collision avoidance. Radar data from ARS408-21 (Continental AG, Hanover, Germany) and cameras (Sony IMX219) were processed using YOLO v5. In [32], Kim et al. proposed a radar-based object detection and classification approach integrating YOLO for accurate detection, achieving 46.16% precision. Radar sensors from Continental AG, Hanover, Germany, were used in the experiments. Additionally, Refs. [33,34] enhance vehicle orientation and tracking using vision detection and CNN, improving detection in complex environments. In ref. [33] the cameras used were Sony IMX477, manufactured by Sony Corporation, Tokyo, Japan, and the LiDAR sensor was Velodyne HDL-32E, produced by Velodyne Lidar, Inc., San Jose, CA, USA. The YOLO v5 model and TensorFlow v2.11 (Google LLC, Mountain View, CA, USA) were employed for detection and data processing. Ref. [34] the radar used was the ARS408-21 sensor, manufactured by Continental AG, Hanover, Germany, and the vision data were processed using OpenCV v4.5 (Intel Corporation, Santa Clara, CA, USA). Caesar et al. [35] tested a 3D LiDAR solution that achieved 65.41% precision in simulations using Velodyne HDL-32E LiDAR, manufactured by Velodyne Lidar, Inc., San Jose, CA, USA, demonstrating its reliability for real-time ADAS applications. Wang et al. [36] introduce RODNet, a radar-and-camera-based algorithm using a 3D auto-encoder, improving ADAS object detection. YOLO v5 was also used for real-time classification. Robsrud et al. [37] combine LiDAR and mmWave radar for improved perception across different ranges, ensuring safer navigation. The system utilized Velodyne VLP-32C LiDAR and ARS408-21 radar sensors, both processed using NVIDIA DriveWorks SDK v3.5 (NVIDIA Corporation, Santa Clara, CA, USA). Yeong et al. [38] review sensor fusion techniques, emphasizing the benefits of multi-sensor fusion in challenging conditions. Lin et al. [39] enhance SLAM (Simultaneous Localization and Mapping) for lightweight vehicles via sensor fusion and real-time mapping. Their setup integrated Velodyne LiDAR data with cameras from Sony Corporation, Tokyo, Japan, for improved real-time mapping via ROS Noetic.

### 2.5. Summary of Sensor-Based Approaches

Sensor-based approaches to autonomous vehicle collision avoidance are evolving rapidly, with each type of sensor contributing unique strengths to improve safety, reliability, and overall system performance. From Table 1, we conclude the following.

Cameras (monocular, CMOS), when combined with deep learning techniques like YOLO and CNN, offer high object detection accuracy, particularly in well-lit conditions. Cameras are cost-effective and provide rich visual data for environmental perception. However, their performance is limited by lighting conditions (e.g., low-light environments) and line-of-sight obstructions, which can hinder their effectiveness in certain scenarios, such as nighttime driving or inclement weather.

Radar excels in adverse weather conditions, such as rain, fog, or snow, and performs well in low-light environments, making it ideal for detecting objects at a distance in challenging conditions. However, radar requires substantial computational power and large datasets to accurately process and interpret real-time data, limiting its real-time detection capability in some applications.

LiDAR provides precise 3D mapping of the environment, enabling accurate obstacle detection and localization. It is particularly useful for mapping complex surroundings and creating detailed 3D models of the environment. However, LiDAR systems are computationally expensive and often require extensive data processing, particularly in dynamic environments where real-time updates are critical.

Sensor fusion: Combining data from multiple sensors—such as LiDAR, radar, and cameras—enhances overall accuracy by compensating for the limitations of individual sensors. While sensor fusion improves detection reliability and robustness, it also increases the system’s complexity and computational demand. This requires sophisticated algorithms and high-performance computing resources to process the combined data efficiently in real time.

In conclusion, cameras, radar, LiDAR, and sensor fusion are widely utilized in real-world autonomous driving systems due to their complementary strengths. These sensors, when integrated effectively, create a robust perception framework that enhances collision avoidance capabilities. Techniques such as deep learning models and advanced sensor setups are primarily employed in simulations to improve algorithm performance and prepare them for real-world deployment, ensuring that autonomous vehicles can safely navigate diverse and dynamic environments.

**Table 1:** Comparison of sensor-based approaches in autonomous vehicles.

Sensor	Approach	Strengths	Limitations
Camera	Ref. [16] VLC-based (Visible Light Communication) positioning using monocular camera	Fast, low-latency communication, cost-effective	Requires line-of-sight, sensitive to lighting conditions
	Ref. [17] Synthetic dataset to train vision algorithms for autonomous driving	Customizable for various conditions (weather, lighting)	May not generalize to real-world environments
	Refs. [18,21] Provides synthetic data for training vision models for AVs	Accurate position and orientation estimation	May not generalize to real-world scenarios
	Ref. [19] DL with inverse perspective mapping for vehicle orientation	Effectively predicts collision risks, even in complex environments	High computational demand, requires large datasets
	Ref. [20] VLC for vehicle-to-vehicle tracking	Real-time tracking, low cost	Sensitive to lighting
Radar	Ref. [22] Radar-based solution for vehicle detection in ADAS	Reliable for real-time applications in ADAS	High computational requirements
	Ref. [23] Machine Learning with radar data to enhance vehicle position estimation	Robustness in adverse weather conditions such as rain or fog	Requires large datasets and computational resources
	Ref. [24] A data-driven method to improve radar accuracy for vehicle position estimation	Better accuracy compared to conventional radar estimation methods	Real-time application and dataset dependency
	Ref. [25] Deep learning-based radar for collision avoidance	Accurate detection using DL	High computational requirements
LIDAR	Ref. [26] Point cloud map generation and localization using 3D LiDAR scans for autonomous vehicles	Highly accurate 3D mapping, useful for real-time localization in AVs	High computational cost, large datasets in dynamic environments
	Ref. [27] Object detection for autonomous vehicles using YOLO algorithm with sensor-based technology	High detection accuracy, fast processing times for object detection using YOLO	Performance depends on the data quality, affected by adverse weather
	Ref. [28] 3D LIDAR for real-time obstacle detection and tracking	Accurate dynamic obstacle detection	High computational demand, large storage and processing capacity
Fusion Sensor	Refs. [30,31] Fuses LIDAR depth data with camera visuals for real-time vehicle detection and 3D object tracking	Combines depth and visual data for better accuracy	Requires complex fusion algorithms, high computational cost
	Ref. [32] Real-time obstacle detection using YOLO model	Fast detection, effective for small objects	Performance drops in low-light conditions
	Ref. [33] Cross-modal supervision for radar and vision object detection	Combining radar and vision-based data for object tracking	High computational demands, potentially limiting its efficiency in real-time applications
	Ref. [34] Use CNNs to improve vehicle detection performance	Relevant for precise navigation and collision avoidance	High computational cost, large datasets required
	Ref. [35] 3D LIDAR point cloud fusion with image segmentation	High accuracy in object localization	Computational complexity, high data processing demands
	Ref. [36] Cross-modal supervision combines radar and vision for object detection	Leverages supervision to improve accuracy and robustness	Requires large datasets for training, high computational cost
	Ref. [37] Combines LIDAR and millimeter-wave radar for robust navigation and collision avoidance	Effective in low-visibility environments, combines depth and radar data	High processing demand due to radar and LIDAR fusion

## 3. Collision Avoidance Techniques

### 3.1. Overview of Collision Avoidance Algorithms

Collision avoidance systems are vital for the safety and functionality of autonomous vehicles (AVs), enabling them to detect and respond to potential hazards in real time. These systems rely on sophisticated algorithms that can be classified into three broad categories: path-planning algorithms, decision-making methods, and Machine Learning approaches. Each category plays a crucial role in ensuring safe, efficient, and reliable vehicle operation.

Path-Planning Algorithms

Path-planning algorithms are responsible for determining safe, efficient, and collision-free routes for AVs to follow. Traditional methods, such as A* and Dijkstra’s Algorithm, are highly effective in well-defined, structured environments where the map and obstacles are known in advance. However, these methods struggle in dynamic or unstructured settings. To address these limitations, advanced techniques like Rapidly Exploring Random Tree (RRT) and its variants have been developed. These methods are particularly well suited for environments with moving obstacles and uncertain road conditions, enabling vehicles to explore and navigate in real time. In recent years, Reinforcement Learning (RL) approaches have emerged, further enhancing path planning. These models allow AVs to adapt and learn optimal routes based on their interactions with the environment, making them more flexible and responsive to unforeseen challenges.

Decision-Making Methods

Decision-making algorithms are designed to handle the tactical choices an AV must make in complex traffic environments. These decisions include lane changes, merging onto highways, yielding at intersections, and responding to the behavior of other road users. Rule-based approaches offer predictable and reliable solutions in well-structured environments but lack the flexibility needed in more complex, real-world situations. For dynamic decision-making, optimization-based methods, such as Model Predictive Control (MPC), are widely used. MPC allows for the optimization of vehicle behavior while considering various constraints, such as speed limits, traffic rules, and safety margins. For highly dynamic environments, Machine Learning (ML) techniques, like Monte Carlo Tree Search (MCTS) combined with RL, offer greater adaptability. These methods enable AVs to make real-time decisions in multi-agent scenarios, where the behavior of other vehicles and pedestrians must be predicted and responded to quickly.

Machine Learning Approaches

Machine Learning plays a pivotal role in enhancing the perception, learning, and adaptation capabilities of collision avoidance systems. Supervised learning is commonly applied to tasks such as object detection and classification, where AVs are trained to recognize and categorize objects in their environment, such as pedestrians, other vehicles, and traffic signals. While supervised learning excels in environments with well-labeled data, it faces challenges in dynamic scenarios with unseen objects. To address this, unsupervised learning and semi-supervised learning techniques are employed to detect anomalies and patterns without the need for exhaustive labeled datasets. Finally, Reinforcement Learning allows AVs to dynamically adapt to changing environments by learning from trial-and-error interactions. Hybrid models that integrate RL with traditional planning and decision-making methods create a robust system capable of tackling a wide range of real-world challenges, from navigating through busy city streets to driving in adverse weather conditions.

By combining these complementary techniques, collision avoidance systems are able to ensure safer, more efficient operation of autonomous vehicles. The following sections will delve deeper into the details of these methodologies, exploring their advantages, limitations, and potential for future development.

### 3.2. Path Planning Algorithms

Path planning algorithms are crucial for helping autonomous vehicles navigate complex environments by detecting obstacles, updating paths in real time, and enabling multi-agent coordination. They evaluate safety restrictions and employ predictive models to enhance the dependability and safety of autonomous driving technology [40].

#### 3.2.1. Path Planning: Classical Approaches

##### A Algorithm*

A* (A-star) is a heuristic search technique that integrates estimated and actual costs to identify the shortest path between two locations. Maw et al. [41] introduced the improved Anytime Dynamic A* (iADA*) for dynamic environments, which modifies real-time pathways in response to impediments. While iADA* is faster than similar algorithms, it generates longer paths and requires further validation. The Traversability Hybrid A* (THA*) algorithm was introduced by Thoresen et al. [42] to optimize path distance using traversability estimation, outperforming hybrid A* over short distances, though it has high computational demands. Alternative methodologies, such as the amalgamation of A* with a low-level controller for the evasion of vulnerable road users (VRUs) [43], and the Hierarchical Long-Term and Short-Term Planner (LTSTP) [44], present promising solutions but necessitate additional testing in more intricate environments. In reference [45], Liu and Zhang introduced an enhanced A* algorithm focused on energy consumption (IA*FC) for the idle states of the ADS. The IA*FC approach demonstrates better performance than the traditional A* algorithm by 16.949% under red-light traffic conditions.

##### Dijkstra’s Algorithm

Dijkstra’s algorithm, created by Edsger W. Dijkstra, solves the single-source shortest path problem by exploring all possible routes from a starting node to all others in a network and updating distances to find the shortest path. Chen et al. [46] combine RRT for initial planning with Dijkstra’s algorithm for optimization, improving real-time performance by 22% on semi-structured roads compared to traditional methods. Zhu and Sun [47] introduced the Reverse Labeling Dijkstra Algorithm (RLDA), which uses reverse labeling. RLDA converged faster than ACO (Ant Colony Optimization), Gs (Genetic Algorithms), and neural network accelerator (NNA) but had longer processing times with larger node counts.

##### Rapidly Exploring Random Tree (RRT)

The Rapidly Exploring Random Tree (RRT) is a popular sampling-based path-planning algorithm known for its probabilistic completeness. Several studies use the Rapidly Exploring Random Tree (RRT) method to focus on path-planning approaches. Wang et al. [48] developed Neural RRT*, a learning-enhanced version of RRT* designed to improve path effectiveness and adaptability in dynamic environments. In addition, Li et al. [49] combine the Improved RRT* algorithm and Artificial Potential Field (APF) to address path planning challenges. Feraco et al. [50] implemented RRT for local trajectory control and planning in autonomous cars, prioritizing stability during navigation. Wang et al. [51] proposed a bi-directional RRT with branch pruning, incorporating kinematic constraints to reduce computational load and enhance efficiency in constrained environments. Huang and Ma in [52] presented an improved RRT algorithm, refining traditional RRT for increased adaptability and effectiveness in autonomous vehicle path planning across varied road conditions. Zhang et al. [53] presented the Improved Adaptive RRT algorithm. As a foundation of the RRT, the sampling-based method was proposed in various papers. For example, Rong et al. [54] developed an attention-based sampling technique that optimizes focus on relevant regions, improving efficiency in complex environments. Jin et al. [55] proposed a sampling-based method for unstructured environments, enhancing robustness and adaptability for autonomous navigation in challenging terrains. These RRT-based methods extend the RRT framework for better performance in real-world autonomous applications.

##### Probabilistic Roadmap (PRM)

The PRM algorithm is a motion planning technique that forms a roadmap of the configuration space by fusing locations through random sampling to find a collision-free path [56]. Lazy PRM delays collision checks until necessary, reducing computation time and adapting efficiently in complex environments [57]. LSPP (Line Segment Path Planning) combines RRT and ADAPF (Azimuth Distance Artificial Potential Field) for obstacle detection and optimization techniques, outperforming algorithms like RRT and PRM, though results rely on graphical analysis [58]. PRM is also highlighted for generating smooth paths in AGVs (Autonomous Ground Vehicles) [59], ensuring collision avoidance in complex environments [60], and optimizing multi-agent systems with evolutionary algorithms [61]. Rakita et al. [62] introduce probability-informed trees in a single-query setup, using probabilistic sampling to reduce computational load while focusing on probable paths.

##### Dynamic Window Approach (DWA)

The Dynamic Window Approach (DWA) is a real-time local path planning method that selects the best trajectory by analyzing the vehicle’s dynamic constraints. Yeong et al. [63] introduced a hybrid A*-PDWA approach, which uses A* for path estimation and P-DWA for obstacle avoidance. Although successful in avoiding collisions, it has not been compared to other methods. Additionally, the IA*-DWA method integrates Bezier curves with A* and DWA to achieve smoother paths, outperforming traditional A* and DWA [64]. The A*-DWA hybrid approach combines A* with Adaptive DWA for real-time trajectory planning, though further parameter optimization and comparisons are needed [65]. Liu et al. [66] employed DWA for local and Dijkstra for global path planning but focused on hardware implementation without statistical performance comparison.

##### Artificial Potential Fields (APF)

The Artificial Potential Field (APF) method forms collision-free paths by generating engaging points toward the objective and offensive forces from obstacles but may struggle with regional minima. Lu’s ADPF-PP (APF for Path Planning) improved on standard APF but still risks local optima in complex environments [67]. The MPC-APF (Model Predictive Control and APF) method, employing curve fitting, performed better than traditional APF but required more validation [68]. Li’s APF-MPC-DS (APF, MPC, driving style) algorithm factored in the driving style for improved stability over APF-MPC, though only graphical data were provided [69]. The CDT-APF method combined APF with constrained Delaunay triangulation, showing enhanced performance, but real-time testing is pending [70]. Zhang’s IAPF-GDM, integrating Gradient Descent, outperformed others but did not handle dynamic obstacles [71]. Li’s DynEFWA-APF for dynamic scenarios did not surpass other algorithms like A*, APF, or GA-APF [72]. Wang’s PCAPF combined APF with polynomial curve optimization to produce effective paths but was not compared to other methods [73].

More details about the path-planning classical approaches can be found in Table 2.

#### 3.2.2. Path Planning: Machine and Deep Learning Technique

Autonomous vehicles (AVs) utilize path planning to prevent collisions; Machine Learning (ML) and deep learning (DL) approaches have been employed to enhance the performance of these systems. These methods assist AVs in planning their routes while avoiding obstacles, taking into account real-time environmental data and making intelligent decisions to ensure safe navigation.

##### Deep Supervised Learning Techniques

Kicki et al. in [74] developed a neural-network-based path planning method for ADS using a gradient-based self-supervised learning algorithm, achieving faster results than RRT* with 74% accuracy. The NNNOC method, based on optimal control theory, performed well in simulations but needed to be faster for real-time applications. Guo et al. proposed an LSTM network trained with fuzzy control, which outperformed existing methods but lacked adaptability to dynamic environments [75]. For End-to-End learning, Lee and Liu [76] introduced DSUNet-PP, enhancing lane centering but suffering from slow processing times. Deep learning techniques, such as convolutional neural networks (CNNs), recurrent neural networks (RNNs), and feedforward neural networks (FFNNs), form the basis of deep learning techniques. CNNs are used for processing images, FFNNs are utilized for supervised learning applications, and RNNs are employed for sequential data. These networks can be implemented in systems like Advanced Driver-Assistance Systems (ADS) to process sensory data, including images [77]. Deep supervised learning methodologies can provide precise and rapid outcomes in well-known contexts. Nevertheless, constructing resilient models necessitates a considerable training dataset. The significant drawbacks of neural networks are the demand for offline training and the laborious retraining process for model changes. A deep cascaded neural network called IVGG-LSTM, introduced by Song et al., surpassed previous networks in terms of effectiveness. Although the IVGG-LSTM network can adapt to different road conditions and learn from human drivers, improvements are still needed for real-world road applications and algorithm performance [78]. Yang and Yao [79] presented the PRRT-BSC, a hybrid method that integrates the Pruning Rapidly Exploring Random Tree (PRRT) with B-Spline Curves. The objective is to identify pathways utilizing a pruning strategy informed by obstacle distribution, followed by applying B-spline interpolation to provide smoother trajectories. Both strategies have produced promising results but have not been compared to others in the literature. Moraes et al. developed Deep Path, a CNN-based driving system, but offered no comparisons to other methods [80]. Sakurai et al. [81] created a Spiking Neural Network (SNN) method in a separate study. This algorithm uses two agents to avoid obstacles and pursue the goal. Using SNN features, these agents can interact and learn from dynamic objects through graphs. The SNN method performed better than the threshold-based agent algorithm. Nonetheless, further validation of the system with more complex obstacles is necessary. Kalathi et al. used neural networks for road sign recognition without path planning [82]. The CNN-Raw-RNN method proposed by Wang et al. for trajectory generation outperformed previous approaches but requires more comparison with other AI methods [83].

##### Reinforcement Learning Techniques (RL)

This section discusses recent advancements in Reinforcement Learning (RL) for autonomous driving systems (ADSs). RL excels in handling complex problems but faces challenges in balancing exploration and exploitation, parameter tuning, and managing large state-action tables [84]. Wang et al.’s AAIRL algorithm enhances AIRL with semantic rewards, improving performance over baseline methods [85]. Chen et al. developed a Q-learning and greedy selection hybrid, outperforming K-shortest and Dijkstra algorithms but lacking support for unknown environments [86]. Kim [87] and Chang [88] each applied Q-learning variations for path planning, showing improved results, though further testing with dynamic obstacles is needed. Low et al.’s IQL algorithm enhanced Q-learning, surpassing RRT and VG in many cases but struggling with dynamic barriers [89]. Liu et al. [90] and [91] integrated RL with A* and PSO, showing superior performance but requiring more validation. Rousseas et al. combined AHPF and RL, outperforming RRT*, but still needs to be applied to dynamic or 3D scenarios [92].

##### Deep Reinforcement Learning Techniques (DRL)

In DRL includes actor–critic, value-based, and policy-based methods, which require more complex parameter tuning and the careful balancing of exploration and exploitation.

##### Value-Based/Policy-Based Methods

The main objective of Reinforcement Learning is to find the best course of action. This involves understanding policy-based and value-based approaches and how value and policy are connected. The Bellman operator (Q-function) extracts value from the policy. The algorithm continually enhances the policy and assesses its value. Reinforcement Learning (RL) aims to find optimal actions using value- and policy-based methods. In autonomous driving systems (ADSs), RL methods like Q-learning are widely used for path planning [93]. Zhao used DDQN with SUMO for collision-free paths, though alternative algorithms were not compared [94]. A hybrid combining Deep Q-Learning and Potential Field improved performance in unknown environments [95], while the Conditional Deep Q-Network (CDQN) enhances path planning but faces obstacle avoidance challenges [96]. Wen’s Deep Q-Networks for junctions shows promising results, but further testing is needed [97].Li’s IDQNPER-ETE works well in static settings but requires dynamic testing [98], while Peng’s DRL-GAT-SA algorithm performs well but needs real-world validation [99]. Perez’s DDPG outperformed DQN in simulations but was not tested on real-world maps [100]. In [101], the authors improved the conventional Hierarchical Double Deep Q-learning (hDDQN) with an LSTM network layer to interact with the surroundings and identify noise. Also, to emulate the ADS system as an End-to-End driving scheme, [102] designed a hybrid neural network architecture. The Deep Reinforcement Learning algorithm learns the DQN network, which handles path planning.

##### Actor–Critic Methods

The actor–critic algorithm combines value-based and policy-based methods. Wang’s HRL-MPC system outperformed SAC and MCQ in path planning but needs testing in complex scenarios [103]. Zhang’s LSAC-CPP algorithm, which improves SAC using collision prediction, performed well but lacks real-world testing [104]. Xu’s ACRL algorithm showed smoother paths than rule-based systems but struggled with new scenarios due to its pre-trained network reliance [105]. Choi’s MCAL-P, based on SAC, was better at avoiding collisions but could not drive straight [106]. Tang’s SAC method outperformed DQN and DDPG in simulations but lacks real-world validation [107].

#### 3.2.3. Path Planning: Meta-Heuristic Optimization Technique

Lately, meta-heuristic optimization techniques have gained popularity for autonomous vehicle (AV) path planning due to their ability to optimize travel time, fuel efficiency, and obstacle avoidance. The Genetic Algorithm (GA), introduced in 1975, uses crossover and mutation to refine solutions. The hybrid GA-PF algorithm [108] combines GA for global path planning with the Potential Field (PF) for local paths but lacks real-world applicability due to missing dynamic modeling. Particle Swarm Optimization (PSO), introduced by Kennedy and Eberhart, has been adapted for AVs. Zhang et al. [109] improved PSO for faster convergence, though it struggles in large, 3D environments. Similarly, Improved PSO (IPSO) [110] is promising for indoor static settings but unsuitable for outdoor dynamic conditions. Ant Colony Optimization (ACO), presented by Dorigo in 1997, mimics ants’ behavior in finding the shortest path. Hybrid algorithms combining ACO with A* [111], PSO [112], and Dijkstra [113] improve performance but face challenges in dynamic environments. In addition, Pohan et al. [114] apply a combination of rule-based and heuristic approaches for autonomous navigation and collision avoidance. The Improved Simulated Annealing (ISA) algorithm [115] reduces execution time for high-dimensional problems. The Artificial Bee Colony (ABC) algorithm, given by Karaboga in 2005, has been enhanced with the Arrhenius ABC [116], improving exploration and exploitation, though further testing is needed. Co-GLABC [117], combining ABC with Differential Evolution (DE), performs better but requires dynamic scenario testing. The Firefly Algorithm (FA) [118] has also been used in AV path planning, with the self-adaptive population size FA (SPSFA) [119] showing improved results but slower performance. Fusic et al. [120] executed Satellite PSO (SPSO) and five PSO variants for path optimization using satellite images, with SPSO underperforming and facing criticism for ignoring other meta-heuristic techniques. Zhang et al. [121] presented AIACSE (Improved ACS algorithm using the population information entropy), an improved ACS with entropy adjustments, excelling over RAS, PS-ACO, and ACS but needing improvement in dynamic settings. The PSOFS method, a combination of the PSO and Fringe Search algorithms, was presented by Wahab et al. [122] for path planning in indoor environments.

#### 3.2.4. Summary of Path Planning

Path planning algorithms for autonomous collision avoidance are critical to autonomous vehicle technology. Several advanced methods have been explored. A* and iADA* are heuristic search algorithms used for dynamic path adjustments but tend to produce longer paths. THA* optimizes path distance but has high computational costs. Further, Dijkstra’s algorithm finds optimal paths that are computationally heavy. RLDA improves convergence speed; however, it struggles with large datasets. Also, RRT, PRRT-BSC, and Attention-RRT* focus on smooth path generation. Nevertheless, further real-world validation is needed. PRM minimizes collision checks for efficiency, and while DWA is effective in real-time trajectory planning, it still requires more comparisons with other methods. On the other hand, APF generates paths using attractive and repulsive forces, but improved versions like IAPF still need help with local minima. Deep Learning Techniques like DSUNet-PP, LSTM, and CNN-Raw-RNN perform well in simulation. Nonetheless, they need better real-world adaptability. In addition, Reinforcement Learning methods like Q-learning and DDQN show promise; however, they need further testing in dynamic environments to balance exploration and exploitation. Finally, from Table 2, Table 3 and Table 4, we conclude that while many algorithms perform well in simulation, they need more real-world testing to ensure their effectiveness in dynamic conditions.

### 3.3. Decision-Making Strategies

**Figure 3:**
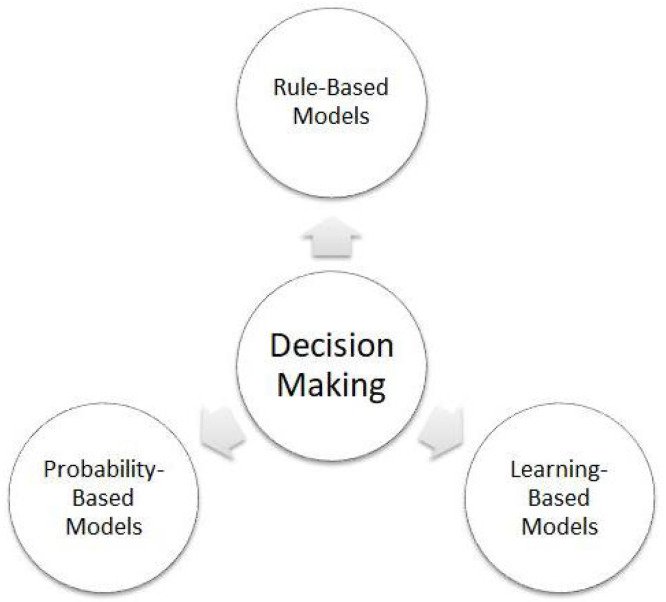
Framework of dsecision-making algorithms.

The rise of autonomous vehicles (AVs) necessitates advanced decision-making strategies for effective collision avoidance Figure 3. As AVs operate in increasingly complex environments, robust decision-making systems are critical [123]. The papers with the best performance in this field are cited and detailed in Table 5.

#### 3.3.1. Rule-Based Models

Rule-based models make decisions based on predefined conditions from expert knowledge or optimization. Zheng et al. [124] utilized the concept of least action, a modification of Lagrange’s equations, to represent the local driving environment as a spring-damped system and to find optimal resolutions for lateral and vertical accelerations. Fuzzy logic models like [125] use expert data to evaluate variables. Further, Xin et al. [126] examined pedestrian behavior at signalized crossings utilizing trajectory data and a decision-tree methodology. Zhang [127] employed a decision tree for the optimal strategy search and risk evaluation of cars at a junction. Hang et al. [128] adapted the parameters of a game theory profit model by incorporating driving characteristics as a variable to account for different driving styles.

#### 3.3.2. Probability-Based Models

Probability-based models use data distributions for decision-making, including Markov processes and Bayesian theory. In AVs, decision-making is often modeled as a Markov process, where each decision depends on the previous state. Sun [129] introduced a modified obstacle mutual collision avoidance (MORCA) prediction model for agent vehicle trajectory prediction, while Sun et al. [130] introduced a lane-changing strategy using the random forest algorithm.

#### 3.3.3. Learning-Based Models

Deep Learning (DL), a subset of Machine Learning, uses bidirectional propagation to adjust parameters until convergence. It is used in autonomous vehicle systems but is still developing for intelligent decision-making in congested traffic [131]. Challenges include replicating experienced driver behavior and understanding environmental semantics. Some DL methods have shown progress, Reinforcement Learning (RL), a branch of DL, models decision-making as a Markov process, training networks using reward functions within an environment [132]. Hoel et al. [133] combined DRL with Monte Carlo Tree Search, similar to the AlphaGo Zero algorithm, for learning and planning in AV decision-making. In addition to Reinforcement-Learning-based behavioral decision-making methods, support vector machines [134] are frequently employed to facilitate action decision-making. Xu et al. [135] presented a Reinforcement Learning methodology for autonomous decision-making in intelligent vehicles on routes. The approach conceptualizes the sequential decision-making challenge of highway change and surpassing as a Markov decision process with considerable objectives, including safety, velocity, and smoothness. Deep Reinforcement Learning (DRL) has been utilized for the selection of optimal driving behaviors [136,137,138,139,140,141].

**Table 5:** Comparison of decision-making models for autonomous vehicles.

Model	Approach	Application	Key Findings
Rule-Based	Ref. [124] Behavioral Decision-Making based on Driving Risk	Risk assessment of intelligent vehicles in real-time driving scenarios.	Developed a model based on Lagrange’s equations to assess driving risks and propose optimal lateral and vertical accelerations for CA
	Ref. [125] Fuzzy Risk Assessment	Risk assessment with interval numbers and assessment distributions.	Developed a fuzzy inference system to address uncertainties and improve vehicle safety in complex environments.
	Ref. [126] Decision Tree based on Trajectory Data	Prediction of pedestrian behaviors at intersections.	Applied gradient boosting decision trees to predict pedestrians’ decisions at signalized intersections, enhancing vehicle safety.
	Ref. [127] Risk-aware Decision-Making	Planning at uncontrolled intersections.	Used a strategy tree to guide vehicles through uncontrolled intersections with risk-aware decision-making.
	Ref. [128] Game Theoretic Decision-Making	Non-cooperative decision-making in autonomous driving.	Incorporated driving characteristics into a non-cooperative game-theoretic model for decision-making.
Probability-Based	Ref. [129] Interactive Decision-Making	Left-turning of autonomous vehicles.	Presented an interactive model for left-turning at uncontrolled intersections to reduce collision risks.
	Ref. [130] Random-Forest-based (RF) Lane Change Strategy	Intelligent driving systems for lane change recognition	Utilized an RF algorithm for lane change strategy analysis, improving decision-making accuracy in lane-changing scenarios.
Learning-Based	Ref. [132] Self-learning Optimal Cruise Control	Cruise control decision-making based on individual driving	Applied self-learning techniques to optimize cruise control decisions based on car-following styles, improving driving performance.
	Ref. [133] DRL and Planning	Tactical decision-making for autonomous driving	Combined DRL with planning algorithms to improve tactical decision making.
	Ref. [134] Mixed Strategy Nash Equilibrium	Autonomous driving at uncontrolled intersections	Proposed a decision-making framework based on intention prediction and mixed strategy Nash equilibrium for safer and efficient navigation
	Ref. [135] Reinforcement Learning (RL)	Autonomous decision making on highways	Developed an RL approach for autonomous decision making on highways, improving vehicle performance in lane changes and overtaking.
	Ref. [136] Multi-Objective Multi-Agent Cooperative Decision-Making	Multi-agent decision-making for autonomous vehicles	Introduced MO-MIX, a DRL-based framework for multi objective, multi agent cooperative decision making, enhancing performance in complex environments.
	Ref. [137] DRL	Decision-making at intersections without traffic signals	Proposed decision making models for AVs at unsignalized intersections using DRL to improve traffic efficiency.
	Ref. [138] RL-Based Autonomous Driving	Driving at intersections in CARLA simulator	Applied RL to autonomous driving in intersection scenarios using the CARLA simulator, demonstrating improved decision-making.
	Ref. [139] DRL for Task Transfer	Decision-making in the intersections that do not have traffic signals	Developed a DRL approach for transferring driving tasks in non-signalized intersections, improving decision-making.
	Ref. [140] Risk-Aware Decision-Making with RL	Automated driving at occluded intersections	Proposed a risk-aware high-level decision-making framework using RL for navigating intersections.
	Ref. [141] DRL with Camera Sensors	Automated driving in complex intersections	Introduced a DRL approach based on camera sensor data for driving automation in intersection scenarios.

#### 3.3.4. Summary of Decision-Making Strategies

In conclusion, decision-making methods for autonomous vehicles (AVs), including rule-based, probabilistic, and learning-based approaches like Machine Learning, deep learning, and Reinforcement Learning, offer distinct advantages (see Table 5). Rule-based systems establish foundational navigation and collision avoidance, while probabilistic and Machine Learning models enhance adaptability. Deep and Reinforcement Learning hold promise for handling complex maneuvers, although they still require refinement for dynamic traffic conditions. However, these methods rely heavily on sensor accuracy, so noise or sensor failures can significantly impact reliability, especially in unstructured or adverse environments like bad weather. Limited vehicle-to-vehicle communication also complicates coordination, and ethical dilemmas in unavoidable collisions add further complexity, underscoring the need for ongoing advancements to improve AV safety and responsiveness.

### 3.4. Machine Learning Approaches

Integrating Machine Learning (ML) into collision avoidance systems for autonomous vehicles (AVs) is crucial for enhancing safety and efficiency. Recent AI and ML advancements have produced algorithms that predict and mitigate collision risks in real time, focusing on object detection and collision prevention [142].

#### 3.4.1. Deep Learning, Reinforcement Learning, and Supervised Learning

Advances in deep learning (DL) have led to robust algorithms for obstacle detection and avoidance. For instance, Zhang [143] uses deep Reinforcement Learning (DRL) to optimize resource allocation and enhance AV safety by enabling real-time learning from the environment.

Kuutti et al. [144] propose a modular framework combining high-level decision-making via ML and low-level control via rule-based systems. This hybrid approach improves safety, as critical control functions are managed by established methods, reducing the risks associated with Machine Learning models.

Reinforcement Learning (RL) plays a crucial role in developing effective collision avoidance strategies. Yuan et al. [145] explore reinforced cooperative collision avoidance, using imitation learning and DRL to minimize collisions through data-driven approaches. Yurtsever et al. [146] introduced a hybrid DRL framework for Advanced Driver Assistance Systems (ADASs), while Peng et al. [147] designed an ADS based on a Dueling Double Deep Q-Network (DDDQN), validated using the Open Racing Car Simulator (TORCS).

Merola et al. [148] proposed a Deep Q-Network-based method for pre-accident scenarios, enabling AVs to perform emergency maneuvers. Cao et al. [149] introduced hierarchical reinforcement and imitation learning (H-REIL) to balance safety and efficiency during near-accident situations.

Ongoing research [150,151,152] integrates DRL with conventional methods, improving performance in complex environments [153,154]. Federated DRL has also been proposed for collision avoidance [155].

Finally, Issa et al. [156] combined deep Q-learning with Faster R-CNN for obstacle detection in dynamic environments, demonstrating the potential of these combined technologies to enhance navigation strategies.

More papers and details are provided in Table 6.

#### 3.4.2. Hybrid Learning Approaches

While individual ML techniques like deep learning and Reinforcement Learning are powerful, hybrid approaches combine their strengths to address real-world complexities more effectively. Hybrid algorithms optimize path planning while addressing obstacle avoidance complexities. For example, [157] proposes a Dragonfly–Fuzzy hybrid controller combining bio-inspired algorithms with fuzzy logic to improve AV efficiency, optimizing both path and obstacle avoidance.

At the core of hybrid algorithms is integrating different algorithmic approaches, such as heuristic methods, optimization techniques, and Machine Learning frameworks. For instance, Huang et al. propose a multi-heuristic hybrid A* algorithm that combines Rapid-Exploring Random Tree (RRT*) with optimization methods to improve path planning for autonomous vehicles [158]. This combination allows for more efficient navigation in complex environments by leveraging the strengths of both algorithms, enabling vehicles to make real-time adjustments to their paths while considering potential obstacles. Yang discusses an integrated spatial kinematics-dynamics Model Predictive Control approach that facilitates collision-free tracking for autonomous vehicles [159]. This method enables vehicles to predict future states and make informed decisions based on dynamic environmental factors, thereby enhancing their ability to avoid collisions. Moreover, incorporating Machine Learning techniques into hybrid algorithms has shown promise in improving collision avoidance systems. For example, Liu discusses the development of a pedestrian trajectory prediction system that allows autonomous vehicles to anticipate the movements of pedestrians, thereby enabling proactive collision avoidance strategies [160]. Jiang et al. [161] developed a hybrid ACO-PSO algorithm with environmental feedback that outperformed traditional ACO but was not assessed for low-dimensional tasks. This predictive capability is essential for navigating urban environments where interactions with pedestrians are frequent. This research emphasizes the importance of developing algorithms that adapt to various driving conditions while adhering to ethical standards and enhancing public trust in autonomous vehicle technologies. Gao et al. discuss how hybrid path planning algorithms for uncrewed surface vehicles incorporate collision avoidance regulations and dynamic obstacles, demonstrating the importance of real-time data exchange in optimizing navigation strategies [162]. Similarly, Kim et al. [163] propose an MPC method that utilizes a time-varying and non-uniformly spaced horizon to enhance path-following and collision avoidance capabilities. Also, the Gaussian-Mixture-Model-based online mapping technique proposed by Lee and Woo [164] highlights the potential of probabilistic models in enhancing the situational awareness of autonomous vehicles, enabling them to navigate complex environments more effectively. Advancements in simulation technologies and experimental validations further support the continuous evolution of hybrid algorithms. For instance, the work of Bezerra et al. on deep-Q-network hybridization with extended Kalman Filters illustrates how simulation environments can be utilized to test and refine collision avoidance strategies [165]. Integrating Machine Learning techniques into hybrid algorithms also plays a crucial role in enhancing collision avoidance strategies. [166] explores the use of improved artificial Potential Fields for dynamic path planning in challenging conditions, such as icy and snowy roads. Another study proposed iABC–EP, combining ABC and EP, which produced 5.75% shorter paths than ABC–EP, but it requires further testing in complex environments [167]. In addition, ref [168] proposes a DRL-based approach to improve autonomous vehicle safety for vulnerable road users (VRUs) like pedestrians and cyclists.

#### 3.4.3. Summary of Machine Learning

Advanced methods like deep learning, Reinforcement Learning (RL), and hybrid algorithms improve performance but have specific limitations in autonomous vehicle (AV) deployment (see Table 6, Table 7 and Table 8). Accuracy: Deep learning models (CNNs, RNNs) excel in detecting obstacles and predicting movements but at higher computational costs. Reaction Time: Rule-based models respond faster but lack adaptability in complex scenarios. Generalization: Unlike rule-based methods, which struggle with unpredictability, Machine Learning generalizes better to new situations.

Robustness: Hybrid models combine the strengths of rule-based and learning systems, offering better performance in adverse conditions.

While more accurate and adaptive, Machine Learning models demand more computation and have slower reaction times. Hybrid approaches provide a balanced solution but add complexity and may require more tuning.

### 3.5. Summary of Key Techniques

Path planning, decision-making, and Machine Learning are key components of autonomous vehicle (AV) collision avoidance systems. Path planning generates safe and efficient trajectories using algorithms like A* and Rapidly Exploring Random Tree (RRT) in structured environments, while Reinforcement Learning enhances adaptability in dynamic settings. Decision-making algorithms, such as rule-based and optimization approaches, handle tactical actions like lane changes and multi-agent coordination, excelling in predictable scenarios. Machine Learning methods, including Monte Carlo Tree Search and Reinforcement Learning, provide flexibility for complex traffic conditions. Supervised learning aids in object detection and classification, while Reinforcement Learning adapts to changing environments, and hybrid models combine these techniques for improved performance. Together, these methods create a complementary framework that boosts safety, efficiency, and scalability. Future research will focus on refining their integration to handle edge cases and computational challenges.

## 4. Real-World Applications and Testing

Autonomous vehicle (AV) collision avoidance algorithms are highly sophisticated, but their practical effectiveness can only be validated through real-world applications and tests. Although many of these algorithms perform well in controlled simulations, the complexity of real-world environments, where variables such as unpredictable pedestrian behavior, complex traffic patterns, and adverse weather conditions exist, presents significant challenges. This section will explore real-world tests of AV collision avoidance systems, highlight the challenges faced, and discuss how various algorithms perform in these settings.

### 4.1. Real-World AV Testing Projects

Several high-profile autonomous vehicle projects have been deployed on public roads, offering valuable information on how collision avoidance algorithms operate in real-world settings. Some of the most well-known projects include the following. 1. Waymo (formerly the Google Self-Driving Car Project) has extensively tested its AVs in various urban and rural environments. Waymo vehicles use a combination of deep learning models and sensor fusion techniques (e.g., LIDAR, radar, and cameras) to detect and dodge collisions with other vehicles, obstacles, and pedestrians. 2. Tesla Autopilot uses sensor-based and neural-network-based algorithms to achieve semi-autonomous driving. Tesla’s vehicles rely heavily on cameras and neural networks for object detection and collision avoidance. Tesla’s software also leverages real-world driving data from its extensive fleet, which enables continuous improvement through deep learning models. 3. Baidu Apollo is an open-source autonomous vehicle platform tested on complex roadways in China, including congested urban areas. Apollo integrates Reinforcement Learning with traditional decision-making techniques for collision avoidance in high-traffic environments. These projects have contributed significantly to understanding how collision avoidance systems perform in real-world conditions and offer lessons on algorithm effectiveness.

### 4.2. Real-World Performance of Collision Avoidance Algorithms

The practical deployment of collision avoidance systems in AVs highlights the strengths and limitations of the algorithms discussed earlier. Below is a comparison of real-world performance across different algorithm types.

1. Sensor-Based Approaches: Sensor fusion (e.g., LIDAR, radar, cameras) is essential for detecting real-time obstacles. In Waymo’s testing, for instance, LIDAR provides accurate 3D mapping of the environment, enabling the vehicle to detect obstacles even in low-light conditions. However, real-world issues such as sensor blindness due to heavy rain or snow have been reported, affecting detection accuracy. While effective in clear weather, Tesla’s camera-based system faces challenges in low-visibility conditions.
2. Path Planning and Decision-Making: Decision-making algorithms incorporating rule-based and optimization-based techniques have been successfully applied in controlled environments like highways (e.g., Tesla Autopilot’s Navigate on Autopilot). However, these algorithms often need help with unpredictable human behaviors in urban environments with mixed traffic, such as jaywalking pedestrians or erratic drivers. Reinforcement Learning models, like those used in Baidu Apollo, have shown potential in these settings by learning from interaction with the environment but still require extensive training and simulation before deployment on real roads.
3. Machine Learning Approaches: In real-world testing, Machine Learning models and intense learning have been integral. Waymo’s use of convolutional neural networks (CNNs) for image-based obstacle detection has proven effective in various driving conditions. Machine Learning models require considerable amounts of tagged data for training, and their performance can be debased when encountering scenarios that are not represented in the training data. This limitation is particularly problematic in urban settings where unusual events (e.g., a pedestrian running into traffic) may occur.

#### 4.2.1. Real-World Accident Cases

Several real-world incidents have underscored the need for more robust collision avoidance systems. In 2018, a self-driving Uber vehicle struck and killed a pedestrian in Tempe, Arizona. The incident highlighted the limitations of AV systems in responding to unpredictable pedestrian behavior. Tesla Autopilot has been involved in collisions due to limitations in detecting static objects, such as a firetruck parked on a highway. These incidents highlight the challenges of developing accurate object detection algorithms.

Uber’s Self-Driving Testing Incident: A fatal collision in 2018 underscored the need for improved sensor fusion and decision-making to handle edge cases like low-light conditions.

#### 4.2.2. Challenges in Real-World Environments

Although autonomous vehicle collision avoidance systems show promise for real-world use, they encounter challenges such as predicting human behavior and dealing with the effects of weather conditions on sensor-based systems. Real-time decision-making requires algorithms to process large amounts of sensor data quickly. Edge computing has been proposed as a possible solution to reduce latency in high computational demand scenarios, but this remains an area of ongoing research.

### 4.3. Cross-Domain Technologies in Collision Avoidance Methods

Integrating cross-domain technologies has become increasingly essential for enhancing the capabilities of collision avoidance methods in autonomous vehicles. These technologies significantly improve perception, decision-making, and real-time communication, addressing critical challenges such as latency, situational awareness, and adaptability to dynamic environments. In the following, we highlight the role of key cross-domain technologies in collision avoidance.

#### 4.3.1. Edge Computing

Edge computing decentralizes data processing by performing computations closer to the source, such as onboard systems or edge devices near the vehicle, significantly reducing latency critical for real-time collision avoidance systems. By processing sensor data like LiDAR and radar streams locally, edge computing enables instantaneous trajectory adjustments and hazard detection, minimizing the risk of accidents caused by delayed responses. For example, autonomous vehicles with edge devices can make real-time decisions in scenarios like sudden pedestrian crossings or merging traffic on highways, where milliseconds saved by local processing can prevent collisions. Additionally, edge computing allows vehicles to bypass the communication delays associated with cloud systems, ensuring consistent performance even in areas with limited network coverage [169].

#### 4.3.2. V2X Communication

Vehicle-to-Everything (V2X) communication facilitates seamless data exchange between vehicles, infrastructure, pedestrians, and cloud systems, enhancing situational awareness and enabling proactive collision avoidance. Through V2X, autonomous cars can receive early warnings about potential hazards, such as a stalled vehicle around a blind corner, allowing sufficient time to adjust speed and path. For example, cooperative adaptive cruise control (CACC) uses V2X to synchronize braking among multiple vehicles, preventing pile-ups during emergency stops. By extending perception beyond the line of sight and enabling coordinated actions, V2X communication significantly enhances the safety and efficiency of collision avoidance systems in dynamic and complex environments [170].

#### 4.3.3. Virtual Reality (VR)

Virtual reality (VR) offers a controlled and cost-effective platform for training and testing collision avoidance algorithms by simulating diverse and complex driving scenarios. Through VR simulations, autonomous vehicles can be exposed to edge cases such as icy roads, erratic pedestrian behaviors, or severe weather conditions without real-world risks. For instance, CARLA, a widely used open-source urban driving simulator, trains Reinforcement Learning algorithms to handle urban and highway scenarios effectively. By providing a safe environment for iterative testing and improvement, VR accelerates the development of robust collision avoidance systems while reducing the costs and dangers associated with real-world testing [171].

#### 4.3.4. Other Information and Communication Technologies

Advanced technologies like 5G networks, sensor fusion, and distributed computing significantly enhance collision avoidance systems by improving communication, perception, and computational efficiency [172]. For instance, 5G enables ultra-low latency communication, supporting real-time data exchange between vehicles and infrastructure, which is crucial for split-second decision-making in collision-prone situations. Sensor fusion combines inputs from LiDAR, radar, and cameras to enhance object detection accuracy, even under adverse weather conditions such as heavy rain or fog. Additionally, distributed computing allows workloads to be shared across multiple processors, ensuring the scalable and efficient operation of autonomous vehicles. These technologies collectively address the challenges of latency, perception gaps, and computational demands in modern collision avoidance frameworks.

By integrating these cross-domain technologies, collision avoidance methods can achieve greater robustness, scalability, and adaptability, addressing critical challenges in autonomous driving.

### 4.4. Recommendations for Improving Real-World Testing

To improve the performance of AV collision avoidance systems in real-world settings, the following steps are recommended:

1. Integration of Real-World and Simulated Data: Using real-world driving data and simulated environments can help train Machine Learning models more effectively.
2. Edge Computing for Real-Time Processing: Leveraging edge computing for faster data processing can help reduce latency, allowing AVs to react quickly to obstacles and collisions.
3. Multi-Sensor Fusion: Integrating data from multiple sensors (LIDAR, radar, cameras) improves performance in challenging conditions. Refining sensor fusion algorithms is essential for real-world performance.
4. Ethical and Safety Considerations: AV systems must prioritize human safety in decision-making, particularly in ambiguous situations where inevitable collisions occur. Ethical frameworks must be integrated into decision-making to ensure AVs prioritize human life.

### 4.5. Summary

While real-world testing has demonstrated the potential of collision avoidance algorithms, significant challenges remain in ensuring that AVs can operate safely in all environments. By improving sensor capabilities, refining decision-making algorithms, and addressing computational constraints, collision avoidance systems can become more reliable and effective in real-world applications. The lessons learned from current real-world testing efforts offer valuable insights for future research and development in the field.

## 5. Conclusions and Future Direction

### 5.1. Expand on Future Research Directions

Given the evolving nature of autonomous vehicle technology, several areas of future research can significantly improve the safety, reliability, and efficiency of collision avoidance systems.

1. Edge Computing for Real-Time Decision Making: Deep learning and Reinforcement Learning are computationally intensive. Edge computing reduces latency by processing data closer to the source, making it suitable for real-time AV applications. Future research should focus on optimizing edge computing for handling high-volume sensor data.
2. Federated Learning for Data Privacy: Federated learning enables AVs to train models locally, sharing updates instead of raw data, thereby preserving privacy. Research should explore its application in improving collision avoidance algorithms, particularly in adapting to different driving conditions.
3. Improving Sim-to-Real Transfer in RL: The gap between simulated and real-world environments challenges RL in AVs. Future work should improve simulation accuracy and apply domain adaptation techniques to bridge this gap.

### 5.2. Conclusions

The future of autonomous vehicle collision avoidance lies in addressing the current limitations of advanced techniques and embracing emerging technologies. The use of edge computing, federated learning, improved Reinforcement Learning environments, and explainable AI will be critical in overcoming the challenges faced by AV systems today. Moreover, advances in sensor technology will provide more accurate environmental data, enabling AVs to make safer, faster, and more reliable decisions. By focusing on these areas of future research, autonomous driving will move closer to achieving fully reliable, collision-free driving experiences.

## Figures and Tables

**Table 2:** Comparison of path planning: classical approaches algorithms for autonomous vehicles.

Method	Approach	Strengths	Limitations
A*	Ref. [41] iADA*: Anytime path planning for real-time applications	Dynamic replanning, fast response times	Complex implementation compared to standard A*
	Ref. [42] Combines A* with traversability analysis for UGVs	Incorporates terrain difficulty into planning	Higher complexity, requires detailed terrain data
	Ref. [43] Proposes a more efficient and robust A* algorithm	Handles dynamic environments better than standard A*	More complex implementation due to additional heuristics
	Ref. [44] Combines motion planning with hierarchical framework	Effective for structured highway scenarios	Limited by assumptions of structured environments
	Ref. [45] Fuel-Efficient A*:Minimize fuel consumption	Reduces energy usage in vehicles	May increase travel time
Dijkstra	Ref. [46] Combines RRT for initial planning with Dijkstra for optimization	Efficient path planning with improved real-time performance	Complexity increases with larger environments
	Ref. [47] Dijkstra’s algorithm to optimize vehicle path planning	Enhanced safety and optimal route at intersections	Increased computational complexity at complex intersections
RRT	Ref. [48] Learning-based RRT* for optimal path planning	Efficient path selection; adapts well to dynamic changes	May struggle with real-time processing in complex environments
	Ref. [49] RRT* for exploration; APF for obstacle avoidance	Handles dynamic environments; resolves APF local minima	APF’s local minima; path smoothness of RRT*
	Ref. [50] Standard RRT for local trajectory control	Stability in trajectory control during local planning	Limited in handling unexpected obstacles dynamically
	Ref. [51] Bi-directional RRT with pruning for efficient path planning	Enhanced planning efficiency and accuracy	Complex implementation
	Ref. [52] Applies improved RRT for collision-free paths in AVs	Optimized for real-time planning	Computationally heavy in dense environments
	Ref. [53] Adaptive version of RRT for lane-based AV navigation	Efficient in structured lane environments	Limited to well-structured lanes
	Ref. [54] Attention-driven sampling distribution for motion planning	Efficient in complex settings; adaptive sampling focuses	High computational needs due to attention mechanism
	Ref. [55] Sampling-based approach for unstructured environments	Robust in highly unstructured settings	Computationally intensive
PRM	Ref. [58] Proposes a new path planning algorithm using line segment features for perception	Efficient path planning with improved accuracy in dynamic environments	Limited application in highly unstructured environments
	Ref. [59] Generates smooth paths using PRM while avoiding sharp turns	Provides smooth and continuous trajectories	Limited scalability in very large or complex environments
	Ref. [61] Combines PRM with evolutionary optimization for MAS in uncertain domains	Optimizes collision avoidance in dynamic environments	High computational complexity
	Ref. [62] Single-query with probability-informed sampling	Low computation needs; efficient for quick planning	May not be as effective in dense, highly constrained spaces
DWA	Ref. [63] Combines PDWA and A* algorithms for path planning of autonomous surface vessels	Adaptable to dynamic marine environments	Computationally demanding in complex scenarios
	Refs. [64,65] Combines improved A* for global planning with DWA	Improved path efficiency with real-time adjustments	Increased complexity due to combined algorithms
	Ref. [66] Combines Dijkstra for global path planning and DWA	Efficient collision avoidance in dynamic settings	Increased complexity when scaling to large environments
APF	Ref. [67] Applies adaptive Potential Field for path planning in complex driving environments	Adapts to a range of scenarios, effective in complex scenes	Performance drops with high-density obstacles
	Ref. [69] Integrates planning and tracking for autonomous vehicles considering driving styles	Personalizes driving behavior, improves safety	Requires significant computational resources
	Ref. [70] Combines DPF and CDT for safe, personalized motion control	Safe and personalized driving behavior	Computationally demanding, requires large datasets
	Ref. [71] Road-oriented motion planning framework for active collision avoidance	Suitable for structured road environments, enhances safety	Limited application to unstructured or dynamic environments
	Ref. [72] Combines optimization techniques with Potential Field for path planning	Ensures optimal path in complex scenarios	Performance may degrade in dynamic environments
	Ref. [73] Focuses on post-collision motion planning and control for autonomous vehicles	Ensures stability and safety after collisions	Limited to post-impact scenarios, not applicable for general path planning

**Table 3:** Comparison of path planning learning techniques for autonomous driving.

Method	Approach	Strengths	Limitations
Self-Supervised Learning	Ref. [74] Rapid path planning for car-like vehicles in urban environments	Suitable for urban navigation, fast response	Struggles with complex real-time constraints.
LSTM Neural Network	Ref. [75] Local path planning for mobile robots using LSTM	Handles dynamic and changing environments effectively	Requires significant computational power
Deep Learning	Ref. [76] End-to-End lane detection and path prediction for autonomous driving	High accuracy in real-time lane detection	Limited to structured environments
Ensemble Learning	Ref. [77] Solving blind drift calibration problem using ensemble learning	Increases accuracy and robustness	Specific to calibration tasks
Deep Neural Networks	Ref. [78] Real-time path generation for autonomous driving using DNNs	Suitable for real-time applications	Requires large amounts of training data
CNN-Based	Ref. [79] Uses convolutional neural networks for local path planning	Strong adaptability to dynamic environments	High computational requirements for real-time applications
Supervised Learning	Ref. [80] Trajectory planning using a hybrid of supervised learning and nonlinear control	High efficiency in trajectory optimization	Limited adaptability to unstructured environments
Neuromorphic Computing	Ref. [81] Path planning and obstacle avoidance using neuromorphic computing	Low power consumption and high efficiency	Complexity in hardware and implementation
Neural Networks	Ref. [82] Self-driving car using neural networks for path planning	Suitable for dynamic environments	High computational demand
Deep Neural Networks	Ref. [83] Trajectory learning using deep neural networks	Accurate trajectory prediction	Requires extensive computational resources
Inverse RL	Ref. [85] Augmented adversarial inverse Reinforcement Learning	Strong performance in decision making, path planning	Requires extensive training
DRL	Ref. [86] Path planning for vehicle platoons on edge networks	Efficient in real-time applications	Requires specialized hardware
Q-Learning	Ref. [87] Real-time path planning through Q-learning’s strategy adjustment	Improved real-time performance	Learning process can be slow
RL	Ref. [88] Path planning with improved dynamic window approach	High adaptability in unknown environments	High computational overhead
Q-Learning	Ref. [89] Modified Q-learning with distance metric and virtual target for path planning	Efficient in short-distance navigation	Performance decreases in larger environments
A* + RL	Ref. [90] Path selection using A* and Reinforcement Learning	Enhanced the accuracy	Computationally intensive
RL + PSO	Ref. [91] Path planning using a hybrid of Reinforcement Learning and Particle Swarm Optimization	High adaptability in dynamic environments	Complex implementation
RL	Ref. [92] RL Optimal motion planning in unknown workspaces using IRL	Suitable for unknown environments	May require high computational power
DRL	Ref. [94] DRL-based approach for highway driving	Accurate decision making	Limited generalization to other environments
Q-learning	Ref. [95] Combining Deep Q-Learning and Potential Field	Suitable for dynamic environments	Requires extensive training
DQN	Ref. [96] Conditional DQN for motion planning with fuzzy logic	High adaptability in dynamic environments	Limited to structured environments
DRL	Ref. [97] Path planning for active SLAM in unknown environments	Suitable for unknown environments	High computational requirements
DQN	Ref. [98] Improved DQN for path planning	Accurate path planning	Limited in complex environments
DRL	Ref. [99] Planning with DRL and GATs (Graph Attention Networks)	High accuracy in path planning	High computational complexity
DRL	Ref. [100] DRL-based control for autonomous vehicles in CARLA	High control accuracy	Limited to simulation environments
Hierarchical RL	Ref. [101] Trajectory planning using hierarchical RL	Effective for complex trajectories	High computational cost
DL	Ref. [102] Deep learning for autonomous driving systems	Effective in complex scenarios	Requires large training datasets
RL	Ref. [103] Trajectory planning for autonomous vehicles using RL	Efficient trajectory planning	High computational cost in real-time applications
RL	Ref. [104] Motion planning with stability guarantees	Ensures safe motion planning in dynamic environments	Limited flexibility in highly uncertain environments
actor–critic RL	Ref. [105] Actor–critic based learning for decision-making and planning	High efficiency in decision-making	Complex training process
RL	Ref. [106] Dynamic obstacle avoidance with path planning	Adaptive to dynamic obstacles	Performance depends heavily on environment structure
Soft actor–critic	Ref. [107] Decision-making and motion planning on highways using soft actor–critic	Effective in highway scenarios	Limited to structured highway environments

**Table 4:** Comparison of path planning meta-heuristic optimization technique in autonomous vehicles.

Method	Approach	Strengths	Limitations
GA and FPF	Ref. [108] Combines GA for global path planning with the Fractional Potential Field (FPF) for local paths	Effective for trajectory tracking	May not adapt well to highly dynamic environments
Improved PSO	Ref. [109] Mobile robot path planning using localized PSO	Optimized for local path planning	May struggle with global optimization
PSO	Ref. [110] AGV path planning using improved PSO	Enhanced path optimization for AGVs	Performance may degrade in unpredictable scenarios
Ant Colony, MDP	Ref. [111] Smooth trajectory generation in grid-based environments	Effective trajectory generation	Requires further testing in dynamic environments
Ant Colony	Ref. [112] Dynamic path planning for traffic congestion	Efficient in congested environments	Performance may degrade in highly dynamic traffic
Ant Colony, Dijkstra	Ref. [113] AGV path optimization model	Effective in optimizing AGV paths	Limited scalability for large urban systems
RRT and Ant Colony	Ref. [114] Combined Rapidly Exploring Random Trees (RRT) and ant colony algorithms	Enhanced the Path planning for autonomous navigation.	Requires further testing in real-world applications
Simulated Annealing	Ref. [115] Improved algorithm for vehicle routing problem	Efficient in routing large fleets	High computational complexity in large-scale problems
Artificial Bee Colony (ABC)	Ref. [116] Modified ABC algorithm for robot path planning	Effective for obstacle avoidance	Limited to static environments
Global Best Guided ABC	Ref. [117] New guided ABC algorithm for robot path planning	Effective in global optimization	Limited validation in real-world applications
Firefly Algorithm	Ref. [118] Path planning for mobile robots using Firefly algorithm	Optimized for smaller spaces	Limited real-world testing
Firefly Algorithm	Ref. [119] Self-adaptive population size for global path planning	Adaptable to environment changes	Requires fine-tuning for larger environments
PSO Variants	Ref. [120] Satellite PSO (SPSO) and five PSO variants with satellite image input	Explores path planning variations in PSO	SPSO underperformed all variants; lacks consideration of other meta-heuristics
Improved ACO	Ref. [121] Adaptive Improved ACS using entropy (AIACSE)	Integrates information entropy for enhanced population diversity; surpassed RAS, PS-ACO, and ACS	Needs optimization for dynamic environments

**Table 6:** Comparison of machine learning methods in autonomous vehicle collision avoidance.

Ref./Method	Approach	Strengths	Limitations
Ref. [143] DRL	Proposes a DRL-based solution to optimize resource allocation with enhanced security features for autonomous vehicles	Improve both resource efficiency and cybersecurity in AVs, addressing two crucial aspects simultaneously	May require extensive computational resources and real-time data for effective decision-making in complex environments
Ref. [144] Weakly supervised RL	Introduces a safety mechanism with virtual safety cages in a weakly supervised Reinforcement Learning framework for autonomous highway driving	Enables safer highway navigation by enforcing safety zones, reducing collision risk	Limited by its reliance on simulated environments, which may not fully translate to real-world performance
Ref. [145] RL	Proposes a cooperative, Reinforcement-Learning-based approach for collision avoidance between multiple autonomous vehicles	Enhances collision avoidance through cooperative interaction, making it suitable for multi-vehicle scenarios	Complex implementation, with challenges in real-time coordination and potential communication limitations
Ref. [146] DRL	Integrates DRL with model-based planners for automated driving, ensuring safety and efficiency in path planning.	Balances model accuracy with safety and efficiency.	Integration complexity and requires careful tuning for different environments.
Ref. [147] Deep Q-Network	Proposes an End-to-End autonomous driving system leveraging dueling double DQN for optimal decision- making in driving tasks.	Enhanced performance with more stable learning than conventional DQN.	Requires large amounts of data and struggles with rare edge cases.
Ref. [148] RL	Introduces a reinforced damage minimization strategy for autonomous vehicles in critical events, focusing on reducing damage.	Effective damage minimization in collision scenarios.	Limited to extreme scenarios, performance in normal driving is untested.
Ref. [149] RL	Develops an RL-based control system for imitative driving policies in near-accident scenarios, improving decision-making during critical moments.	Mimics human-like behavior in near-accident scenarios.	High complexity in imitative learning and sensitivity to parameter tuning.
Ref. [150] DRL	Focuses on DRL-based driving policy designed for autonomous road vehicles, improving safety and control in real-world scenarios.	Strong policy learning for real-world environments.	Computationally expensive and data-hungry.
Ref. [151] RL	Discusses the reward misdesign problem in autonomous driving and how improper reward functions can negatively affect RL-based driving policies.	Highlights the importance of reward shaping in RL.	Hard to design universally effective reward functions.
Ref. [152] RL	Reviews dynamic state estimation for connected vehicles, improving decision-making under uncertainty.	Effective in handling uncertain environments.	High reliance on accurate state estimation models.
Ref. [153] DRL	Combines heuristic algorithms with DRL for path planning and obstacle avoidance in lunar exploration missions.	Strong adaptability to unstructured environments.	Specialized for space missions; may not generalize to road driving.
Ref. [154] DRL	Proposes a method for reducing oscillations in obstacle avoidance using DRL and time-derivative of an artificial Potential Field.	Improved stability in obstacle avoidance.	May struggle with highly dynamic obstacles.
Ref. [155] Federated DRL	Uses federated DRL for collision avoidance in autonomous vehicles, focusing on collaborative learning from decentralized sources.	Enables learning from distributed data while maintaining privacy.	Computationally expensive and coordination complexity in federated learning.

**Table 7:** Comparison of hybrid learning approaches for autonomous vehicles.

Ref./Method	Approach	Strengths	Limitations
Ref. [157] Dragonfly–Fuzzy Hybrid Controller	Combines Dragonfly Algorithm with Fuzzy Logic for path planning.	Provides a hybrid method for autonomous vehicle path planning in dynamic environments, ensuring smooth and adaptive control.	High computational complexity due to hybridization; may struggle with real-time applications in highly dynamic environments.
Ref. [158] Multi-Heuristic Hybrid A*	Search-based algorithm tailored for autonomous parking in complex scenarios.	Enhances path planning efficiency in autonomous parking by reducing computation time.	The use of multiple heuristics can increase the complexity and computational time; not well suited for real-time applications in highly dynamic scenarios.
Ref. [159] Spatial Kinematics Dynamics MPC	Integrates spatial kinematics and dynamics for collision-free vehicle tracking.	Provides robust tracking control, improving vehicle safety in dynamic environments.	Computationally expensive and difficult to implement in real time; requires precise kinematic and dynamic modeling of the environment.
Ref. [160] Pedestrian Trajectory Prediction System	Real-time pedestrian prediction system based on Jetson Xavier.	Enhances real-time collision avoidance with accurate pedestrian behavior prediction.	High dependence on accurate pedestrian detection and tracking; may face issues in crowded environments with occlusions.
Ref. [161] Hybrid (ACO-PSO)	Hybrid ACO-PSO with environmental feedback	Outperformed traditional ACO, showing better adaptability	Not assessed for low-dimensional problems
Ref. [162] Hybrid Path Planning with Collision Avoidance Regulations	Focuses on unmanned surface vehicle (USV) navigation in inland rivers, incorporating collision avoidance regulations.	Efficient collision avoidance for USVs in dynamic water environments.	Limited adaptability to sudden environmental changes; heavy reliance on pre-set regulations may not cover all possible scenarios.
Ref. [163] Model Predictive Control (MPC)	Utilizes a time-varying, non-uniform horizon for predictive control in autonomous vehicles.	Improves trajectory prediction and control in highly dynamic environments.	Requires precise modeling of the environment, which may be difficult in complex, real-world scenarios; computationally expensive for long horizons.
Ref. [164] Gaussian Mixture Model-Based Online Mapping	Collision avoidance for unmanned surface vehicles (USV) using real-time mapping and navigation.	Reactively avoids obstacles in marine environments with low computational cost.	Struggles with high-speed navigation in cluttered environments due to the time needed for real-time mapping and collision avoidance decisions.
Ref. [165] DQN with Extended Kalman Filter	Hybrid approach combining DQN and EKF for autonomous navigation.	Accelerates learning and improves decision-making in dynamic environments.	Requires significant training data and time; performance can degrade in highly dynamic environments with unpredictable obstacles.
Ref. [166] Improved Artificial Potential Field (APF)	Path planning in icy and snowy road conditions using an enhanced APF model.	Focuses on vehicle safety and control in extreme weather conditions.	Susceptible to local minima, particularly in dense or complex environments; may not perform well in highly dynamic settings with moving obstacles.
Ref. [167] Hybrid (ABC-EP)	Combination of Artificial Bee Colony (ABC) with Evolutionary Programming (EP)	Achieved 5.75% shorter paths than ABC–EP	Further testing needed in complex environments
Ref. [168] Deep Reinforcement Learning (DRL)	Focuses on collision avoidance in autonomous driving with a focus on vulnerable road users (pedestrians, cyclists).	Provides a learning-based system for real-time obstacle detection and collision avoidance, particularly enhancing safety for vulnerable road users.	Requires extensive training time and data; may not generalize well to unseen environments; struggles with real-time response in highly dynamic environments.

**Table 8:** Specific limitations of advanced collision avoidance techniques.

Technique	Specific Limitations
Deep Learning (CNNs, RNNs, LSTMs)	Requires vast labeled datasets, computationally expensive, lacks interpretability (“black box” nature), and struggles with generalization in rare or extreme scenarios.
Reinforcement Learning (RL)	Long training times, challenges in sim-to-real transfer, and potential risks in real-world exploration due to the exploration-exploitation trade-off.
Hybrid Approaches	Increased complexity in integration, potential latency in decision-making, and requires extensive tuning and optimization for smooth performance.
